# Tenatomy

**Published:** 1882-04

**Authors:** J. V. Newton


### VII.—TENATOMY.

REPORTED BY J. V. NEWTON, V.S.

Some time ago I had occasion to see a horse suffering from a disease of the hock joint, which had lasted for more than a year. The hock had been fired and blistered repeatedly, until the final result of the original disease and treatment was in a contraction of the tendons to such a degree that the toe could not reach the ground. At this time he was walking on the fetlock joint.

I gave an unfavorable prognosis. The horse was then cast and both tendons divided, and shod with a long toe shoe, and at the end of three months was able to do his share of farm work. A few months later could be driven on the road, and showed but very little lameness.

TOLEDO, OHIO, Feb. 2, 1882.

